# Specificity of DNA Vaccines against the Genogroup J and U Infectious Hematopoietic Necrosis Virus Strains Prevalent in China

**DOI:** 10.3390/v14122707

**Published:** 2022-12-02

**Authors:** Caiyun Huo, Dandan Huang, Zhihong Ma, Guiping Li, Tieliang Li, Wutong Lin, Na Jiang, Wei Xing, Guanling Xu, Huanhuan Yu, Lin Luo, Huiling Sun

**Affiliations:** 1 Beijing Key Laboratory for Prevention and Control of Infectious Diseases in Livestock and Poultry, Institute of Animal Husbandry and Veterinary Medicine, Beijing Academy of Agriculture and Forestry Sciences, No. 9 Shuguang Huayuan Zhonglu, Haidian District, Beijing 100097, China; 2 Beijing Fisheries Research Institute, Beijing Academy of Agriculture and Forestry Sciences, No. 18 Jiaomen Road, Fengtai District, Beijing 100068, China

**Keywords:** infectious hematopoietic necrosis virus, G gene, DNA vaccine, genogroup U, cross-immune protection

## Abstract

Infectious hematopoietic necrosis virus (IHNV) is the most important pathogen threatening the aquaculture of salmonid fish in China. In addition to the common genogroup J IHNV, genogroup U has been newly discovered in China. However, there is no effective DNA vaccine to fight against this emerging genogroup U IHNV in China. In this study, DNA vaccines encoding the IHNV viral glycoprotein (G) gene of the GS2014 (genogroup J) and BjLL (genogroup U) strains isolated from northern China were successfully developed, which were identified by restriction analysis and IFA. The expression of the Mx-1 gene and G gene in the spleens and muscles of the injection site as well as the titers of the serum antibodies were measured to evaluate the vaccine efficacy by RT-qPCR and ELISA. We found that DNA vaccine immunization could activate Mx1 gene expression and upregulate G gene expression, and the mRNA levels of the Mx1 gene in the muscles were significantly higher than those in the spleens. Notably, DNA vaccine immunization might not promote the serum antibody in fish at the early stage of immunization. Furthermore, the efficacy of the constructed vaccines was tested in intra- and cross-genogroup challenges by a viral challenge in vivo. It seemed that the DNA vaccines were able to provide great immune protection against IHNV infection. In addition, the genogroup J IHNV-G DNA vaccine showed better immune efficacy than the genogroup U IHNV-G or divalent vaccine, which could provide cross-immune protection against the genogroup U IHNV challenge. Therefore, this is the first study to construct an IHNV DNA vaccine using the G gene from an emerging genogroup U IHNV strain in China. The results provide great insight into the advances of new prophylactic strategies to fight both the genogroup J and U IHNV in China.

## 1. Introduction

Infectious hematopoietic necrosis virus (IHNV) is a rhabdovirus belonging to the genus Novirhabdovirus, family Rhabdoviridae, which causes acute infection in salmonid fish and serious economic losses in salmonid farming [1,2]. Epidemics of infectious hematopoietic necrosis (IHN) can cause mortality at rates exceeding 90% in some cases, depending upon the host species, viral strain, and fish-farming environment [1,3]. In China, the first IHNV outbreak occurred in 1985 in the northeast region. This outbreak resulted in the death of 50,000 rainbow trout within 15 days. According to a previous phylogenetic analysis of partial glycoprotein (G) gene fragments or the complete nucleotide sequence of the G gene, the worldwide IHNV strains can be divided into five genogroups U, M, L, E, and J. The genogroup J is the common IHNV genogroup reported in China [4]. In 2012, the transmission of the genogroup U IHNV into China was confirmed for the first time by our group, named BjLL [5]. This new strain was isolated from north China and has significantly lower virulence than that of the genogroup J IHNV isolate GS2014 from northwest China. Thus, the appearance of this new IHNV genogroup U makes the epidemic status of rainbow trout more complex and more difficult to control.

To reduce the economic losses caused by this pathogen, various candidate IHNV vaccines have been developed, including attenuated vaccines, killed virus, and vaccines based on recombinant DNA technology [6,7,8,9]. However, these traditional vaccines have not provided the ideal protection for rainbow trout [10]. DNA vaccines could overcome many limitations associated with traditional methods of vaccination. The first experiment showing an immune response to plasmid-encoded antigens of infectious pathogens was published several years ago [11,12,13,14]. This very promising technology immediately caught the widespread attention of scientists working in the field of DNA vaccine development. DNA vaccines have been developed for a wide variety of viruses, including influenza virus, human immunodeficiency virus, rabies virus, hepatitis B virus, and rubella virus [15,16,17,18,19]. DNA vaccines have the potential to elicit significant responses in many species, such as larger animals, which include ferrets, pigs, cattle, and nonhuman primates [20]. For aquatic organisms, DNA vaccines also offer several advantages over classical antigen vaccines, and there has been great interest in using this technology to develop vaccines for aquaculture animals [21]. From a practical point of view, they are relatively inexpensive and easy to produce, and all DNA vaccines require the identical production process [22]. In addition, DNA is a very stable molecule and does not need to be maintained in a cold environment during shipment or storage. Importantly, DNA-based immunization also has immunological advantages over traditional methods of vaccination, which can induce strong humoral and cellular immune responses without the risk of inadvertent infection. Considering the above advantages, DNA vaccines have been developed to prevent and control IHNV. For example, E.D. Anderson et al. were the first to construct plasmid vectors encoding the IHNV G gene under the control of a cytomegalovirus promoter and found that the vaccinated fish can be protected from a subsequent IHNV challenge [23]. S. Corbeil et al. demonstrated that a single dose of 1–10 ng of pcDNA-3.1-G gene vaccine can protect rainbow trout fry against a waterborne challenge by IHNV [10]. In addition, a novel suicidal DNA vaccine containing two operons was developed by Marta Alonso et al. The immune effect of this suicidal DNA vaccine is enhanced by activating IHNV M gene expression and inducing apoptosis of the invading cells [24]. In 2005, the APEX-IHN DNA vaccine encoding the G gene was approved for commercial production in Canada [25]. In China, previous studies have reported the protection efficacy of constructed IHNV DNA vaccines based on the G gene of the genotype J IHNV strain isolated from rainbow trout [26]. However, the protection efficacy of DNA vaccines can be different based on the genogroup of the infecting IHNV strain. With the emergence of the new genogroup U IHNV in China, it is necessary to establish a DNA vaccine to fight against this genogroup.

In the present study, three kinds of DNA vaccines were constructed based on the G gene of the genogroup J IHNV strain (GS2014) and genogroup U IHNV strain (BjLL) prevalent in China, one of them being a bivalent vaccine of both strains. The immunogenicity and protective effectiveness of these DNA vaccines against homologous and heterologous IHNV infection after vaccination were determined. To our knowledge, this is the first study to construct an IHNV DNA vaccine using the G gene from an emerging genogroup U IHNV strain in China that demonstrates for the first time the great cross-immune protection between genogroup J and U IHNV DNA vaccines in China. The results provide great insight into the advances of new prophylactic strategies to fight against IHN by focusing on both genogroup J and U IHNV-G DNA vaccines in China.

## 2. Materials and Methods

### 2.1. Viral Strains and Cell Lines

IHNV GS2014 (genogroup J) and IHNV BjLL (genogroup U) were laboratory stocks in Beijing Academy of Agriculture and Forestry Sciences. The human embryonic kidney cells 293T used for plasmid transfection were also reserved in Beijing Academy of Agriculture and Forestry Sciences.

### 2.2. Construction of DNA Vaccine

The G genes of IHNV GS2014 and IHNV BjLL were cloned into the PMD19-T vector and inserted into eukaryotic plasmid pcDNA-3.1 (+) vector using HindIII and XhoI to construct the recombinant plasmid pcDNA-GS2014 and pcDNA-BjLL, respectively, according to a previous report [27]. All vaccine plasmids and the vector plasmid pCDNA3.1 were amplified in *Escherichia coli* strain DH5a and prepared with an EndoFree plasmid extract kit (Tiangen, Shanghai, China). The primer sequences of G genes are shown in Table S1. Underlined sequences are cleavage sites of restriction enzymes.

For the bivalent DNA vaccine of pcDNA-GS2014-BjLL, cleavage sites of restriction enzyme of HindIII, BamHI, and XhoI were introduced. Firstly, the G gene of IHNV GS2014 (genogroup J) was cloned into pGEM-T vector and inserted into eukaryotic plasmid pcDNA-3.1 (+) vector using HindIII and BamHI to construct the recombinant plasmid pcDNA-GS2014. Then, the G gene of IHNV BjLL (genogroup U) was cloned into pGEM-T vector using BamHI and XhoI and ligated with pcDNA-GS2014 to construct the recombinant plasmid pcDNA-GS2014-BjLL. The primer sequences of G genes are shown in Table S1. Underlined sequences are cleavage sites of restriction enzyme.

### 2.3. Restriction Analysis for DNA Vaccine

Restriction analysis for pcDNA-GS2014 and pcDNA-BjLL was performed by restriction enzyme digestion. Here, the single-restriction enzyme digestion systems of reaction consisted of 1 μL XhoI (20 U/μL), 2 μL cutsmart 10X buffer, 2 μL plasmid, and 15 μL water. The double-restriction enzyme digestion systems of reaction consisted of 0.75 μL HindIII (20 U/μL), 0.75 μL XhoI (20 U/μL), 2 μL 10X NEBuffer r2.1, 2 μL plasmid, and 14 μL water. After water bath at 37 °C for 3 h, the enzyme-digested products were identified by agarose gel electrophoresis.

In terms of the bivalent DNA vaccine of pcDNA-GS2014-BjLL, the single-restriction enzyme digestion systems of reaction consisted of 1 μL BamHI (20 U/μL), 2 μL cutsmart 10X buffer, 2 μL plasmid, and 15 μL water. The double-restriction enzyme digestion required three steps, and the reaction systems of each step were as follows: (1) 1 μL BamHI (20 U/μL), 1 μL XhoI (20 U/μL), 2 μL 10X NEBuffer r3.1, 2 μL K buffer, 1.5 μL plasmid, and 15.5 μL water; (2) 1 μL HindIII (20 U/μL), 1 μL BamHI (20 U/μL), 2 μL 10X NEBuffer r3.1, 1.5 μL plasmid, and 15.5 μL water; (3) 1 μL HindIII (20 U/μL), 1 μL XhoI (20 U/μL), 2 μL 10X NEBuffer r2.1, 1.5 μL plasmid, and 14.5 μL water. Finally, these enzyme-digested products were also identified by agarose gel electrophoresis.

### 2.4. Indirect Immunofluorescence Assay (IFA)

IFA was used to measure the G gene expression of the above DNA vaccines. Briefly, 293T cells were maintained in Dulbecco’s modified Eagle’s medium (DMEM) (Hyclone) supplemented with 10% fetal bovine serum (FBS) (Gibco). Subsequently, 1 × 10^6^ cells were plated into 24-well plates coated with polylysine and incubated at 37 °C, 5% CO_2_ overnight. For transfection, 0.75 μg recombinant DNA plasmid and 2 µL Lipofectamine 2000 reagent (Invitrogen) were diluted in 50 µL minimum essential medium (MEM) (Gibco) and incubated at room temperature for 5 min and then were mixed and incubated at room temperature for 20 min. After 20 min, 400 µL mixture was added into the 24-well plate with 400 µL MEM. After 4–6 h transfection, the culture medium was replaced with 500 μL DMEM containing 1.5% FBS and cultured for 24 h in a 5% CO_2_ incubator at 37 °C. Transfected cells were fixed with 4% formaldehyde and blocked with 5% skim milk. Then, cells were incubated with a rabbit anti-IHNV-glycoprotein polyclonal antibody as the first antibody for 12 h at 4 °C and fluorescein-isothiocyanate (FITC)-conjugated goat anti-rabbit-IgG antibody as the secondary antibody for 1 h at room temperature. Observation could be performed under an inverted fluorescence microscope. Here, the expression and purification of anti-IHNV-glycoprotein polyclonal antibody were performed according to previous studies [26,28].

### 2.5. Immunization of DNA Vaccine in Rainbow Trout

Before the beginning of trial, fish were fed with the commercial rainbow trout feedstuff (Beijing Hanye Science and Technology Co., Beijing, China) for two weeks to acclimatize them to the experimental conditions. In order to ensure nearly constant and optimal water quality for fish, the experimental conditions were controlled at temperature 13 ± 1 °C. Then, 2 trials were performed as follows:

Trial 1: A total of 90 healthy fish (mean initial weight 5 ± 1 g; mean initial length 7.5 ± 0.5 cm) were randomly distributed to one control and two vaccine groups (pcDNA-GS2014 and pcDNA-BjLL). Rainbow trout were intramuscularly immunized with 1 μg/100 μL DNA vaccine in the dorsal fin, and the control fish were treated with phosphate-buffered saline (PBS) alone. The serums were collected at days 1, 4, 7, 14, 21, 28, and 35 (three fish per group each time) after immunization and stored at −80 °C. The spleens and muscles of the injection sites were collected at days 1, 4, 7, 14, 21, and 28 (three fish per group each time) after immunization and stored at −80 °C.

Trial 2: A total of 1680 healthy fish (mean initial weight 5 ± 1 g) were randomly distributed to 28 groups. Groups 1–8 were intramuscularly immunized with 1 μg/100 μL pcDNA-BjLL in the dorsal fin. Groups 9–16 were intramuscularly immunized with 1 μg/100 μL pcDNA-GS2014 in the dorsal fin. Groups 17–20 were intramuscularly immunized with 1 μg/100 μL pcDNA-GS2014-BjLL in the dorsal fin. Groups 21–28 were intramuscularly treated with PBS alone. At indicated times after immunization, viral challenge in vivo was performed as follows.

The handling of the animals followed the Guidelines of the Animal Care and Use Committee of Animal Husbandry and Veterinary Medicine of the Beijing Academy of Agriculture and Forestry Sciences (IAHVM-BAAFS).

### 2.6. Determination of Viral G Gene and Mx1 Gene Expression by Real-Time Quantitative PCR (RT-qPCR)

The mRNA levels of viral G gene and Mx1 gene were determined by RT-qPCR. Briefly, total RNA was extracted from approximately 10 mg of spleen or muscle tissue homogenized in TRIzol reagent (Invitrogen), and cDNA was reverse-transcribed. For RT reactions, the first reaction system consisted of 3 μL total RNA (100 ng/μL), 3 μL random primer (50 μM), and 15 μL water. After water bath at 70 °C for 10 min and ice bath for 2 min, the above template RNA was added to the second reaction system that also included 6 μL 5X M-MLV buffer, 1.5 μL dNTP mixture (10 μM), 0.75 μL RNase inhibitor (40 U/μL), and 0.75 μL RTase M-MLV (200 U/μL) and was performed as follows: 30 °C 10 min; 42 °C 1 h; and 70 °C 15 min. Then, the obtained cDNA was amplified. The qPCR reaction systems consisted of 5 μL 2X SYBR Green, 0.2 μL forward primer (10 μM), 0.2 μL reverse primer (10 μM), 2 μL cDNA, and 2.6 μL water. Amplification was performed as follows: 94 °C 2 min; 94 °C 45 s; 57 °C 45 s; 72 °C 25 s (40 cycles); and 72 °C 10 min. The copy number of the G gene was calculated using a G-containing plasmid of known concentration as a standard. Here, the PMD19T-BjLL-G or PMD19T-GS2014-G was used as the standard plasmid, for which copy concentrations were 6.074 × 10^10^ copies/μL. During the experiments, the standard plasmid was serially diluted from 10^−1^ to 10^−8^. For Mx1 gene, the gene expression was normalized to the results of the PBS mock-vaccinated control group using the 2^−ΔΔCT^ method with ARP as a reference gene. The primer sequences are shown in Table S1.

### 2.7. Detection of Serum Antibodies

To determine the serum antibodies in immunized fish, the double-antibody sandwich enzyme-linked immunosorbent assay (ELISA) was adopted in accordance with the instructions of the fish IHNV Ab ELISA kit (TSZ, Lexington, MA, USA). Firstly, the testing sample was diluted 5 times. Subsequently, the wells were added with 50 μL diluted testing sample, negative control, and positive control. After incubation at 37 °C for 30 min, the wells were washed 5 times. Then, the wells were added with 50 μL HRP-conjugate reagent. Similarly, the wells were incubated at 37 °C for 30 min and washed again. Finally, the wells were stained by adding 50 μL chromogen solution A and 50 μL chromogen solution B, and then the reaction was stopped by 50 μL stop solution. The absorbance was determined at 450 nm.

### 2.8. Viral Challenge In Vivo

To assess the immune effects of constructed DNA vaccines in rainbow trout, virus challenge of IHNV was performed. After trial 2 as mentioned above, viral challenge in vivo was performed in immunized fish (mean weight 7.2 g) by batch immersion in 1 L of static water for 1 h at 13 °C. For groups 1–4, 9–12, and 21–24, rainbow trout were challenged with BjLL at dose of 10^5.8^ TCID50/mL at days 4, 7, 21, or 35 after immunization. For groups 5–8, 13–16, and 25–28, rainbow trout were challenged with GS-2014 at dose of 10^5.8^ TCID50/mL at days 4, 7, 21, or 35 after immunization. For groups 17–18, rainbow trout were challenged with BjLL at dose of 10^5.8^ TCID50/mL at days 7 or 35 after immunization. For groups 19–20, rainbow trout were challenged with GS-2014 at dose of 10^5.8^ TCID50/mL at days 7 or 35 after immunization. At 28 days postinfection (dpi), cumulative percentage mortality (CPM) and relative percent survival (RPS) were calculated for all groups. Here, the calculation formula of RPS was RPS = 100%—CPM in DNA vaccine group/CPM in control group.

### 2.9. Data Analysis

Data analysis was conducted using two-way analysis of variance with GraphPad Prism (ver. 5.0). *p* < 0.05 represents statistical significance. The results are shown as the means ± standard deviations of three independent experiments.

## 3. Results

### 3.1. Identification of DNA Vaccine

**Figure 1:**
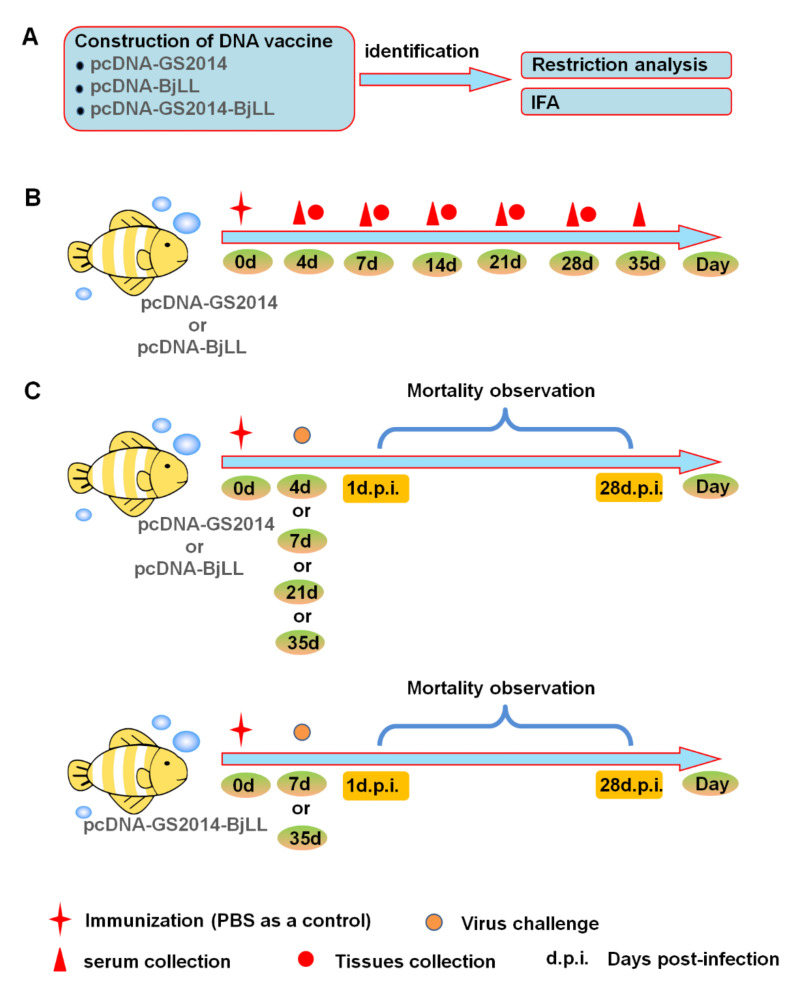
Experimental design schematic. (**A**) Three DNA vaccines were constructed by cloning the G gene of IHNV isolates GS2014 (genogroup J), BjLL (genogroup U) into the pcDNA3.1 vector, and a bivalent vaccine of pcDNA-GS2014-BjLL. Constructed DNA vaccines were identified by PCR and restriction analysis as well as IFA. (**B**) Rainbow trout were immunized with two DNA vaccines at dose of 1 μg, and then the spleens and muscles of the injection site were collected at days 1, 4, 7, 14, 21, and 28 after immunization. In addition, serums were collected at days 1, 4, 7, 14, 21, 28, and 35 after immunization. (**C**) Rainbow trout were challenged with BjLL or GS2014 at days 4, 7, 21, and 35 after immunization of pcDNA-BjLL or pcDNA-GS2014. In addition, rainbow trout were challenged with BjLL or GS2014 at days 7 and 35 after immunization of pcDNA-GS2014-BjLL, respectively. The morbidity and mortality of rainbow trout were observed and recorded during 28 days after challenge.

**Figure 2:**
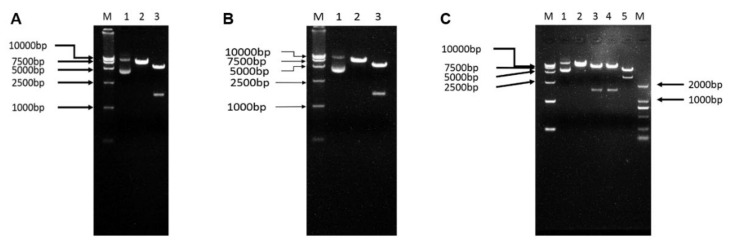
Restriction analysis for DNA vaccine. (**A**,**B**) Restriction analysis for pcDNA-BjLL and pcDNA-GS2014, respectively. M: DL15000 marker; 1: plasmid; 2: single-restriction enzyme digestion by XhoΙ; 3: double-restriction enzyme digestion by HindIII and XhoΙ. (**C**) Restriction analysis for bivalent pcDNA-GS2014-BjLL. M: DL15000 marker; 1: plasmid; 2: single-restriction enzyme digestion by BamHI; 3: double-restriction enzyme digestion by BamHI and XhoΙ; 4: double-restriction enzyme digestion by HindIII and BamHI; 5: double-restriction enzyme digestion by HindIII and XhoΙ; M: DL2000 marker.

To evaluate whether constructed DNA vaccines were successful, the plasmids were extracted and identified as outlined in Figure 1A. These three DNA vaccines were subjected to a restriction analysis for further identification. As shown in Figure 2A,B, enzyme-digested products were about 7000 bp in size by single-restriction enzyme digestion for pcDNA-GS2014 and pcDNA-BjLL, respectively. In addition, 5428 bp and 1527 bp bands could be observed in enzyme-digested products by double-restriction enzyme digestion for these two kinds of DNA vaccines. In terms of the bivalent DNA vaccine of pcDNA-GS2014-BjLL, there was approximately an 8500 bp band via single-restriction enzyme digestion (Figure 2C). Additionally, double-restriction enzyme digestion could obtain four bands at the size of 7000 bp and 1527 bp or 5428 bp and 3060 bp. Thus, all these sizes of the fragments were also consistent with the expected fragments.

**Figure 3:**
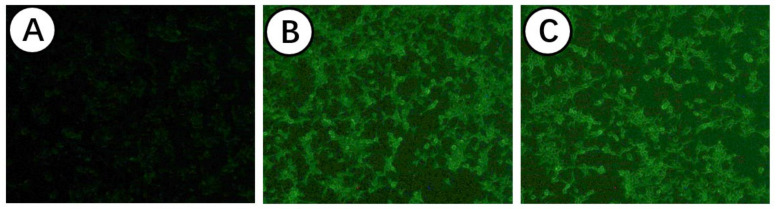
Expression of IHNV-G protein of DNA vaccine in 293T cells by IFA. Recombinant DNA vaccine plasmids were transiently transfected into 293T cells, and the expression of IHNV-G protein was detected by IFA. (**A**) Nontransfected cells. (**B**) Cells transfected with pcDNA-BjLL. (**C**) Cells transfected with pcDNA-GS2014.

In addition, these recombinant eukaryotic plasmids were transiently transfected into 293T cells, and the expression of the IHNV-G protein was detected by IFA. As displayed in Figure 3, there was no fluorescence signal in the nontransfected 293T cells. However, specific green fluorescence could be observed in the cells following transfection with the DNA vaccine plasmids, indicating the in vitro expression of the IHNV-G protein of our DNA vaccines. Above all, the results demonstrate that these DNA vaccines are successfully constructed and can be applied in the following experiments.

### 3.2. DNA Vaccine Immunization Activates Mx1 Gene Expression

**Figure 4:**
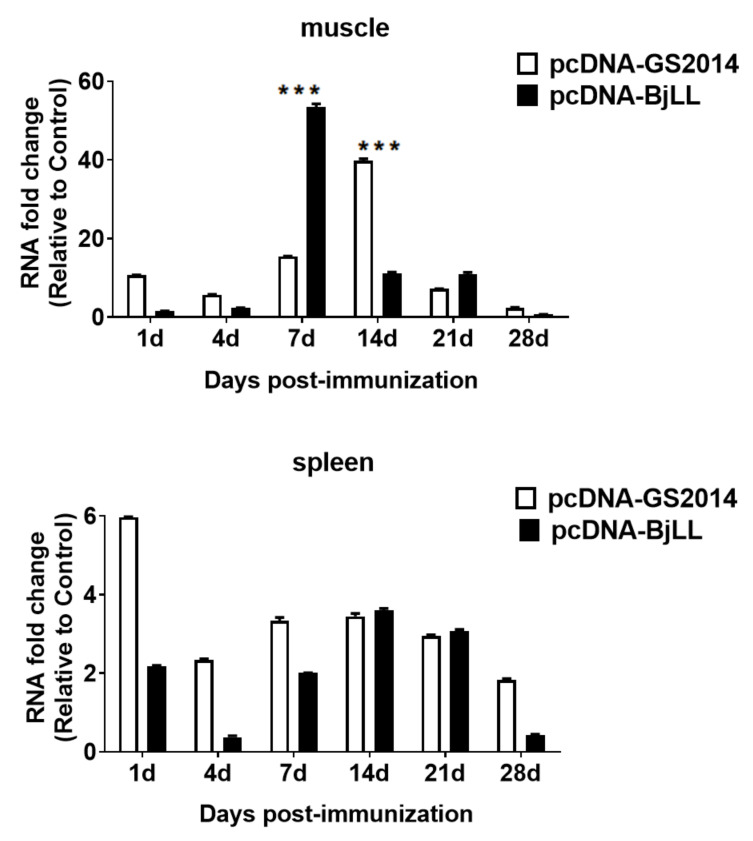
Relative expression of Mx1 gene postvaccination. The Mx1 gene expression in spleen and muscles of the injection site at days 1, 4, 7, 14, 21, and 28 after immunization were measured by RT-qPCR. The data of immunization group were compared with that of PBS control group. Graphs are shown from three independent replicates (*** *p* < 0.001).

The Mx1 gene encodes interferon-inducible proteins that confer nonspecific resistance against viruses in different mammalian species. The expression of the Mx1 gene was determined by RT-qPCR assay to reveal a potentiation of nonspecific antiviral defenses in fish after injection of the DNA vaccines. As outlined in Figure 1B, rainbow trout were immunized with two DNA vaccines at a dose of 1 μg, and then the spleens and muscles of the injection site were collected at days 1, 4, 7, 14, 21, and 28 after immunization. Comparing the Mx1 expression in different organs of rainbow trout immunized with the same IHNV DNA vaccines, the mRNA levels of the Mx1 gene in the muscles were significantly higher than those in the spleens (Figure 4). The expression of Mx1 in the spleens had no significant difference among the DNA vaccination groups and the control group. In the muscles, the Mx1 gene expression of the pcDNA-BjLL group was gradually upregulated from days 1 to 7 and reached a peak at day 7. In terms of pcDNA-GS2014, the mRNA values of this gene were gradually increased from days 1 to 14 following immunization and reached a peak at day 14. By comparison of these two DNA vaccines, there were significant differences in muscles at days 7 and 14 after immunization (*p* < 0.05). Taken together, the Mx1 gene is therefore activated by the intramuscular DNA vaccine injection in rainbow trout.

### 3.3. DNA Vaccine Immunization Increases G Gene Expression

**Figure 5:**
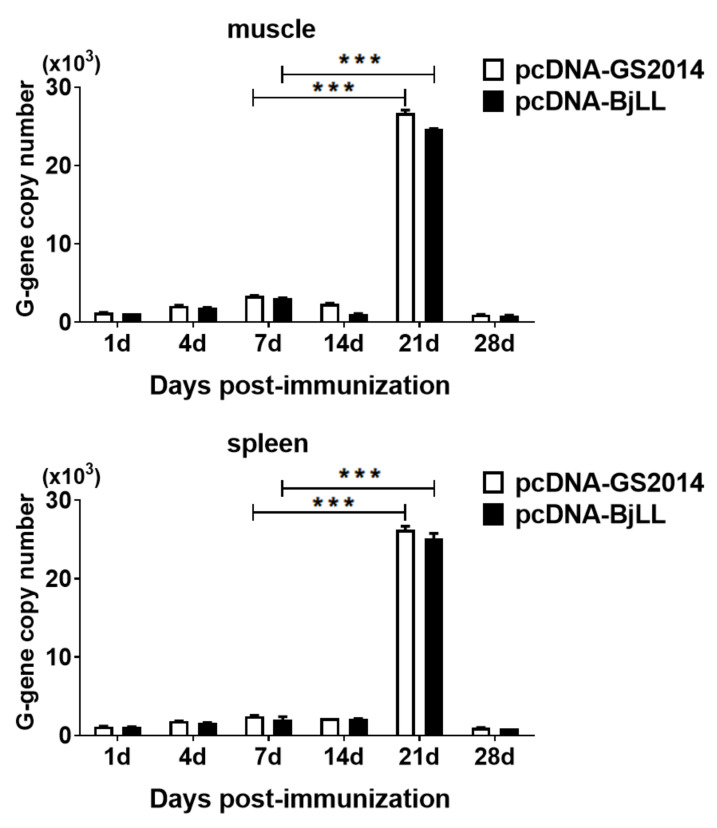
Expression of G gene postvaccination. The G gene expression in spleen and muscles of the injection site at days 1, 4, 7, 14, 21, and 28 after immunization were measured by RT-qPCR. Graphs are shown from three independent replicates (*** *p* < 0.001).

In order to detect the G gene expression in the tissues of fish following immunization, an RT-qPCR assay was adopted as outlined in Figure 1B. Similarly, rainbow trout were immunized with two DNA vaccines, and then the spleens and muscles of the injection site were collected at days 1, 4, 7, 14, 21, and 28 after immunization. As shown in Figure 5, the G gene expression in both the spleens and muscles reached a peak at day 21 after immunization, which was dramatically higher than that at day 7 (*p* < 0.05). At day 28, the expression tended to be decreased. It seemed that there was a cycle of rising and falling over the entire timeline with intervals of 7 days. Therefore, the results indicate that DNA vaccine immunization can increase viral G gene expression in fish, which facilitates the induction of immune protection.

### 3.4. The Effects of DNA Vaccine on Serum Antibodies at the Early Stage of Immunization

**Figure 6:**
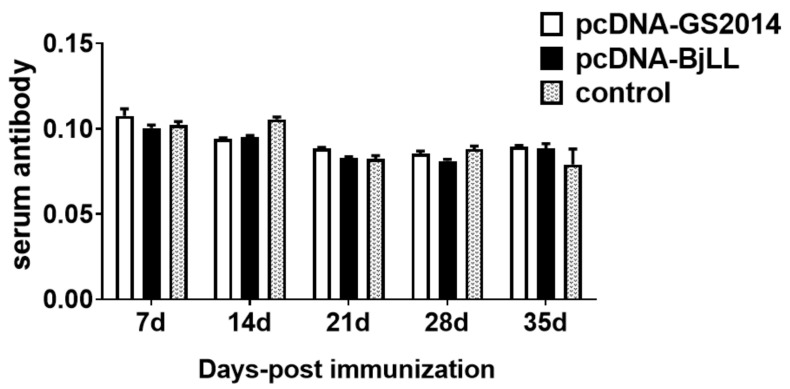
Detection of NAbs in serum. The NAbs in serum at days 7, 14, 21, 28, and 35 after immunization were measured by ELISA. Graphs are shown from three independent replicates.

At days 7, 14, 21, 28, and 35, sera from three fish vaccinated with the DNA vaccine (without viral challenge) were tested. The serum antibodies were determined for each of the groups in separate assays and are shown in Figure 6. Regardless of pcDNA-GS2014 or pcDNA-BjLL, the antibodies in the serum of the fish immunized with the DNA vaccine showed no difference compared with the control group. Together, the results confirm that DNA vaccine immunization might not promote the serum antibody in juvenile rainbow trout during the 35-day immunization period.

### 3.5. DNA Vaccine Has a Strong Protective Efficacy on Rainbow Trout to Fight against IHNV

**Figure 7:**
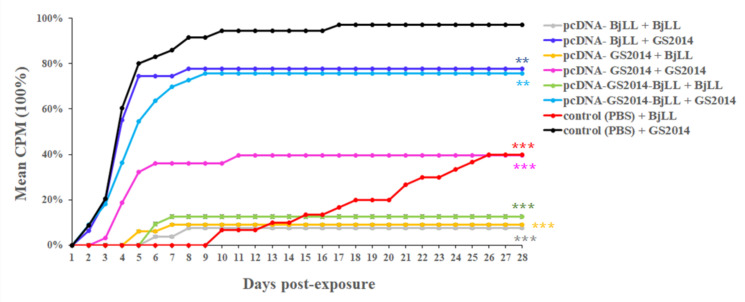
Mean cumulative mortality curves from the challenge experiments. At day 35 after immunization of DNA vaccines, rainbow trout were challenged with BjLL or GS2014 at dose of 10^5.8^ TCID50/mL by batch immersion. The fish were monitored for 28 days postinfection (dpi), and cumulative percentage mortality (CPM) was calculated for all groups. The data of each group were compared with that of control (PBS) + GS2014 group. (** *p* < 0.05; *** *p* < 0.001).

To obtain a better understanding of the genogroup-specific aspects of protection induced by IHNV DNA vaccines, the efficacy of constructed vaccines was tested in intra- and cross-genogroup challenges. As outlined in Figure 1C, rainbow trout were challenged with BjLL or GS2014 at days 4, 7, 21, or 35 after immunization. The morbidity and mortality of rainbow trout were observed and recorded during the observation period of 28 days after the challenge. A total of 28–47% and 91–97% of the fish in the control groups were dead following the viral challenge of the IHNV strains BjLL and GS2014, respectively (Table 1). Thus, the genogroup J IHNV had a much higher lethality in rainbow trout than in the genogroup U IHNV. If the viral challenge was taken at days 4, 7, or 21 after immunization, the pcDNA-BjLL vaccine had no immune protection against the U type IHNV infection, for which the CPM was up to 73–87%. However, the pcDNA-GS2014 and the bivalent pcDNA-GS2014-BjLL vaccine had a certain cross-protection in rainbow trout infected with the genogroup U IHNV, for which the CPM ranged from 24% to 28%. When the viral challenge was performed at day 35 after immunization, the pcDNA-BjLL vaccine showed a high level of immune protection against genogroup U IHNV infection with an RPS of 82% (Figure 7 and Table 1). In addition, this immunization way could also produce cross-protection in rainbow trout infected with the genogroup J IHNV, for which the RPS was 20%. In addition, pcDNA-GS2014 could also provide immunity protection in the genogroup U IHNV-infected rainbow trout with an RPS of 77% and the genogroup J IHNV-infected rainbow trout with an RPS of 60%. Therefore, the genogroup J IHNV-G DNA vaccine showed better immune efficacy than the genogroup U IHNV-G and the divalent vaccine. Above all, the results suggest that the IHNV-G DNA vaccine immunization strategy is protective.

## 4. Discussion

IHN is a major infectious disease endangering the salmon farming industry in China, causing severe economic losses to the Chinese salmon farming business, and it is not friendly to animal welfare and the environment. The first outbreak of IHN occurred in Benxi, northeast China, in 1985, and 600,000 rainbow trout died in 3–15 days on a rainbow trout farm in 1986 [29]. IHN has become the most important pathogen threatening the development of the salmonid fish farming industry in China, and the isolates mainly belong to genogroup J [4]. In 2012, we were the first to report a strain of Chinese IHNV from the outbreak and phylogenetically identify this isolate, named BjLL, which is clustered into the genogroup U according to the phylogenetic analysis of the N gene [5]. Although this new IHNV genogroup in China has significantly lower virulence than that of the genogroup J IHNV isolate GS2014 from northwest China, effective strategies for the prevention and control of IHV caused by these different genogroups of J and U IHNV are urgently needed.

DNA vaccines have several practical and immunological advantages that make them very attractive for the aquaculture industry. DNA vaccination of rainbow trout against IHNV has been demonstrated to be highly efficacious in general, and the vaccine containing M (pM) virus glycoprotein genes has been shown to provide strong protection under various experimental conditions [30,31,32]. In China, great progress has been made in the development of IHNV DNA vaccines against the IHNV of genogroup J. For example, a previous study has shown a DNA vaccine against IHNV genogroup J isolates [26]. This DNA vaccine is constructed by cloning the G gene of the Chinese IHNV isolate SD-12 (genogroup J) into the pcDNA3.1 vector, and the intra-genogroup protective efficacy is determined with diverse IHNV strains from different geographic locations in China. Nevertheless, the protection efficacy of a DNA vaccine can vary based on the genogroup of the infecting IHNV strain. With the emergence of the new genogroup U IHNV in China, there is a need to establish a DNA vaccine to fight against this genogroup in order to decrease economic losses in the fish farming industry. Herein, we developed three kinds of DNA vaccines against not only genogroup J IHNV but also the genogroup U IHNV strains prevalent in China. Similarly, DNA vaccines in this trial were also constructed by cloning the G gene of the Chinese IHNV isolates GS2014 (genogroup J) and BjLL (genogroup U) into the pcDNA3.1 vector. In addition, we also prepared a bivalent DNA vaccine of pcDNA-GS2014-BjLL by cloning the G gene of IHNV BjLL into the pGEM-T vector and then ligating with pcDNA-GS2014. To evaluate whether our constructed DNA vaccines were successful, we identified them by restriction analysis and IFA. Not surprisingly, single-restriction and double-restriction enzyme digestion could obtain the fragments that were in line with expectations. IFA is a method that uses fluorescent antibodies to trace or examine the corresponding antigen. In this study, the recombinant eukaryotic plasmids of the DNA vaccines were transfected into 293T cells, and the expression of the IHNV-G protein was detected by IFA. We could observe green fluorescence in cells following transfection with DNA vaccine plasmids; thus, the IHNV-G proteins of our DNA vaccines were able to be expressed in 293T cells. Hence, DNA vaccines against genogroup J IHNV and genogroup U IHNV strains were successfully constructed.

In order to evaluate the innate immune effects of the above DNA vaccines, rainbow trout were immunized with pcDNA-GS2014 and pcDNA-BjLL. Fish are a lower vertebrate, and their natural immunity is more developed. IFN has a broad spectrum of antiviral activity and participates in immune regulation, which plays an important role in viral replication in fish [33]. Although DNA vaccine or viral infection can induce the expression of IFN or other antiviral genes, there is no effective method to detect IFN in fish directly so far. The Mx protein is an antiviral protein induced by type I IFN [34,35]. When hosts are infected by viruses, this protein can form the antiviral defense line together with other proteins induced by type I IFN. Due to its stable expression and long half-life, the Mx1 protein can be used as a symbol of IFN expression and recognized as a marker of nonspecific immunity induced by a DNA vaccine in fish [32,34]. In rainbow trout, type I IFN has been reported to upregulate Mx expression and mediate early antiviral protection against IHNV [36]. Previous studies by M.M. Penaranda et al. and M.K. Purcell et al. have also validated that the Mx-1 expression fold change is higher in muscles than in the anterior kidney tissues or spleens of rainbow trout using DNA vaccines [32,37]. They also demonstrated that DNA vaccines can upregulate Mx1 gene expression at day 7 after immunization. In our study, a RT-qPCR method was also developed to detect the expression of the Mx1 gene for assessment of the level of nonspecific immunity induced by DNA vaccines. It was the expression of Mx1 in muscles rather than in spleens that significantly changed after immunization of DNA vaccines, indicating that the Mx1 gene in different tissues responds differently to nonspecific immunity stimulated by DNA vaccines. In addition, Mx1 gene expression could reach a peak at day 7 and day 14 in fish following immunization of pcDNA-BjLL and pcDNA-GS2014, respectively. Regardless of the type of IHNV DNA vaccine, Mx1 gene expression was most significantly induced at 7–14 days after immunization, indicating the strong innate immune response. Herein, our results are consistent with previous studies reported by M. Penaranda et al. and M.K. Purcell et al. Notably, the Mx1 gene was downregulated at day 21 postimmunization. It is possible that the level of the innate immune response, as measured by Mx-1 gene expression, can play an important role in the level of the subsequent adaptive response. In a word, pcDNA-GS2014 and pcDNA-BjLL can activate an IFN-dependent innate immune response in rainbow trout.

The G protein is the main antigen of IHNV, and the immune response induced by the DNA vaccine is also related to the expression of the G protein in the body. The G gene expression in tissues of fish following immunization of pcDNA-GS2014 and pcDNA-BjLL was detected. These two DNA vaccines could all upregulate the G gene expression in both spleen and muscle, for which levels reached a peak at day 21. Thus, increased viral G gene expression in fish by these DNA vaccines could contribute to the induction of immune protection. Notably, the G gene expression had a cycle of rising and falling over the entire timeline, with intervals of 7 days. This suggests that the expression of the DNA vaccine antigen G gene in the body was discontinuous with a certain periodicity, and the expression of the G gene and Mx1 gene was not shown consistently.

The specific humoral immunity of fish is mainly involved in immunoglobulins, such as IgM, IgD, and IgZ. Although the mechanism of antibody production in fish is consistent with that in mammals, the difference is that the intensity of antibody immune response in fish can be affected by species and environmental changes. Serum antibodies induced by viral infection or vaccine are an important part of adaptive immunity. Thus, an investigation of the levels and specificity of serum antibodies produced by vaccination provides a functional comparison of the host humoral adaptive immune responses to the DNA vaccines. In our study, the serum antibodies levels were also detected at 7–35 days after DNA vaccine immunization. However, it seemed that the constructed DNA vaccines could not increase serum antibody levels. Thus, DNA vaccine immunization might not promote the serum antibody in juvenile rainbow trout during the 35-day immunization period. Nevertheless, the serum antibody values cannot fully reflect the level of immune protection and may vary with vaccine construction, species, and environmental condition. In addition, it should be noted that the alternative mechanism of nonantibody-dependent immune protection also plays an important role in the resistance to IHNV infection in rainbow trout. Therefore, it is worth further exploring the nonantibody-dependent mechanism behind our results in the future.

To further understand if the enhanced levels of the immune responses induced by the DNA vaccines pcDNA-GS2014 and pcDNA-BjLL as well as bivalent pcDNA-GS2014-BjLL could have the protective efficacy on fish to fight against IHNV, rainbow trout were challenged with BjLL or GS2014 at days 4, 7, 21, or 35 after immunization of pcDNA-BjLL or pcDNA-GS2014. In addition, rainbow trout were also challenged with BjLL or GS2014 at days 7 or 35 after immunization of pcDNA-GS2014-BjLL, respectively. Here, the aim of selecting above four time points for the viral challenge was to find out the shortest time for IHNV-G DNA vaccines to produce complete protection for rainbow trout. After the viral challenge, the genogroup J IHNV had much higher pathogenicity in rainbow trout than genogroup U IHNV, which was just the same as previously reported [5]. In addition, DNA vaccines were able to provide great immune protection against IHNV infection if the viral challenge was taken at day 35 after immunization. Compared with pcDNA-BjLL or pcDNA-GS2014-BjLL, pcDNA-GS2014 had better immune effects and could produce a certain level of cross-immune protection against the genogroup U IHNV challenge. For pcDNA-GS2014-BjLL, this bivalent vaccine might inhibit the ability of pcDNA-GS2014 to induce a host immune response to some extent, and so it affected its immune protection effect alone. In addition, NAbs levels do not appear to correlate with the great protection of DNA vaccines against IHNV, indicating that other adaptive immune mechanisms are likely important in providing protection against IHNV. In short, our IHNV-G DNA vaccines have a strong protective efficacy in rainbow trout to fight against the genogroup J and genogroup U IHNV at day 35 after immunization, especially pcDNA-GS2014.

In conclusion, DNA vaccines were constructed by cloning the G gene of IHNV isolates GS2014 (genogroup J) and BjLL (genogroup U) into the pcDNA3.1 vector. A dose of l μg of the vaccine per rainbow trout (mean weight, 4–6 g) can activate the immune response by regulating the Mx1 gene and IHNV-G expression. These vaccines can provide protection against a challenge with the genogroup J and genogroup U IHNV. Among three DNA vaccines, the genogroup J IHNV-G DNA vaccine not only provided significant protection against the challenge with the parental IHNV strain GS2014, but also provided almost the same cross-protection against the challenge with the emerging genogroup U IHNV strain BjLL. The advantages of these DNA vaccines as antivirals include the fact that they are safe, stable and easily prepared, and they induce robust immune responses as well as protection. To our knowledge, this is the first study to develop a DNA vaccine against the genogroup U IHNV isolate in China and demonstrate for the first time the great cross-immune protection between the genogroup J and U IHNV DNA vaccines in China. The observations demonstrate for the first time that these DNA vaccines play an important role in the comprehensive control of IHN caused by the common genogroup J IHNV and emerging genogroup U IHNV in China.

## Figures and Tables

**Table 1:** Mortality of the immunity protective test.

Groups	Strains for Viral Challenge	Viral Challenge at 4d Post-Immunization		Viral Challenge at 7d Post-Immunization		Viral Challenge at 21d Post-Immunization		Viral Challenge at 35d Post-Immunization	
		CPM	RPS	CPM	RPS	CPM	RPS	CPM	RPS
pcDNA-BjLL	BjLL	87%	-	82%	-	73%	-	7%	82%
pcDNA-BjLL	GS2014	93%	-	81%	16%	93%	-	78%	20%
pcDNA-GS2014	BjLL	28%	13%	33%	30%	24%	14%	9%	77%
pcDNA-GS2014	GS2014	68%	25%	74%	24%	68%	25%	40%	60%
pcDNA-GS2014-BjLL	BjLL	-	-	27%	43%	-	-	12%	69%
pcDNA-GS2014-BjLL	GS2014	-	-	76%	22%	-	-	76%	22%
control (PBS)	BjLL	32%	-	47%	-	28%	-	40%	-
control (PBS)	GS2014	91%	-	97%	-	91%	-	97%	-

## Data Availability

The original contributions presented in the study are included in the article. Further inquiries can be directed to the corresponding authors.

## Supplementary Materials

The following supporting information can be downloaded at: https://www.mdpi.com/article/10.3390/v14122707/s1, Table S1: The primer sequences.
