# Supplementary material for: Specificity of DNA Vaccines against the Genogroup J and U Infectious Hematopoietic Necrosis Virus Strains Prevalent in China
Source: Viruses. 2022 Dec 2;14(12):2707. doi: 10.3390/v14122707 (PMC9780822; doi:10.3390/v14122707)
Supplement: Supplementary file 1 [file viruses-14-02707-s001.zip › viruses-2015866 supplementary.pdf]

Supporting information

Specificity of DNA vaccines against the genogroup J and U infectious hematopoietic necrosis virus strains prevalent in China

Caiyun Huo[1, †], Dandan Huang[1, †], Zhihong Ma[2], Guiping Li[1], Tieliang Li[2], Wutong Lin[1], Na Jiang[2], Wei Xing[2], Guanling Xu[2], Huanhuan Yu[2], Lin Luo[2, *], Huiling Sun[1, *]

[1] Beijing Key Laboratory for Prevention and Control of Infectious Diseases in Livestock and Poultry, Institute of Animal Husbandry and Veterinary Medicine, Beijing Academy of Agriculture and Forestry Sciences, Beijing, China.

[2] Beijing Fisheries Research Institute, Beijing Academy of Agriculture and Forestry Sciences, Beijing, China.

[†] **Contributed to the work equally.**

*** Corresponding author:**

Dr. Huiling Sun, Beijing Key Laboratory for Prevention and Control of Infectious Diseases in Livestock and Poultry, Institute of Animal Husbandry and Veterinary Medicine, Beijing Academy of Agriculture and Forestry Science, No. 9 Shuguang Huayuan Zhonglu, Haidian District, Beijing, 100097, China. Email: sunhuiling01@163.com

Lin Luo, Beijing Fisheries Research Institute, Beijing Academy of Agriculture and Forestry Science, No. 18 Jiaomen Road, Fengtai District, Beijing, 100068, China.

Email: luo_lin666@sina.com

Table S1. The primer sequences

	Target name	Primers
Construction of pcDNA-GS2014 and pcDNA-BjLL	GS2014-G	5’- CCCAAGCTTGCCACCATGGACACCATGATCACCAC -3’
		5’- CCGCTCGAGTTAGGACCTGTTTGCCAG -3’
	BjLL-G	5’- CCCAAGCTTGCCACCATGGACACCATGATCACCAC -3’
		5’- CCGCTCGAGTTAGGACCGGTTTGCCAG -3’
Construction of pcDNA-GS2014-BjLL	GS2014-G	5’- CCCAAGCTTGCCACCATGGACACCATGATCACCAC -3’
		5’- CGCGGATCCGGACCTGTTTGCCAGGT -3’
	BjLL-G	5’- CGCGGATCCGCCACCATGGACACCATGATCACCAC -3’
		5’- CCGCTCGAGTTAGGACCGGTTTGCCAG -3’
Determination of viral G gene and Mx1 gene expression by RT-qPCR	GS2014-G	5’- TCTCAACTGAGATGCCCAAG -3’
		5’- TGTAGTTCCCTTGGGTGTGA -3’
	BjLL-G	5’- TGGAGCAGAAATCCTCTCTCGT -3’
		5’- ATGTGGAGATCGGAACTTGG -3’
	Mx1	5’- GGTTGTGCCATGCAACGTT -3’
		5’- GGCTTGGTCAGGATGCCTAAT -3’
	ARP	5’- GAAAATCATCCAATTGCTGGATG -3’
		5’- CTTCCCACGCAAGGACAGA -3’
